# Photoperiodic Regulation of Flowering Time through Periodic Histone Deacetylation of the Florigen Gene *FT*

**DOI:** 10.1371/journal.pbio.1001649

**Published:** 2013-09-03

**Authors:** Xiaofeng Gu, Yizhong Wang, Yuehui He

**Affiliations:** 1 Department of Biological Sciences, National University of Singapore, Singapore; 2 Temasek Life Sciences Laboratory, Singapore; 3 Shanghai Center for Plant Stress Biology, Chinese Academy of Sciences, Shanghai, China; University of California Riverside, United States of America

## Abstract

The developmental transition from a vegetative to a reproductive phase (i.e., flowering) is timed by the seasonal cue day length or photoperiod in many plant species. Through the photoperiod pathway, inductive day lengths trigger the production of a systemic flowering signal, florigen, to provoke the floral transition. FLOWERING LOCUS T (FT), widely conserved in angiosperms, is a major component of the mobile florigen. In the long-day plant *Arabidopsis*, *FT* expression is rhythmically activated by the output of the photoperiod pathway *CONSTANS* (*CO*), specifically at the end of long days. How *FT* expression is modulated at an adequate level in response to the long-day cue to set a proper flowering time remains unknown. Here, we report a periodic histone deacetylation mechanism for the photoperiodic modulation of *FT* expression. We have identified a plant-unique core structural component of an *Arabidopsis* histone deacetylase (HDAC) complex. In long days, this component accumulates at dusk, and is recruited by a MADS-domain transcription factor to the *FT* locus specifically at the end of the day, leading to periodic histone deacetylation of *FT* chromatin at dusk. Furthermore, we found that at the end of long days CO activity not only activates *FT* expression but also enables HDAC-activity recruitment to *FT* chromatin to dampen the level of *FT* expression, and so prevent precocious flowering in response to the inductive long-day cue. These results collectively reveal a periodic histone deacetylation mechanism for the day-length control of flowering time in higher plants.

## Abstract 2

The seasonal cue day length regulates the timing of the floral transition in plants through periodic histone modifications of the *FT* gene, which encodes a flowering signal in plants. These modifications dampen *FT* expression at dusk to prevent precocious flowering.

## Author Summary

The timing of the developmental transition from a vegetative to a reproductive phase is critically important for reproductive success in flowering plants. Plants synchronize the timing of their floral transition with the changing seasons to flower at a suitable time. The change in day length, or photoperiod, is a key seasonal cue, especially at high latitudes. It is through the photoperiod pathway that day lengths trigger the production of a systemic flowering signal, florigen, to induce the transition from vegetative to reproductive growth and flowering. The FT protein is a major component of the mobile florigen signal. In the model flowering plant *Arabidopsis*, *FT* mRNA expression is rhythmically activated by the gene *CONSTANS*, which is the output of the photoperiod pathway, specifically at the end of long days. In this study, we aimed to address how the level of *FT* expression is modulated in response to the long-day cue to set the appropriate flowering time. We show that on long days a transcription factor recruits histone deacetylase activity to remove acetyl marks from histones at the *FT* gene specifically at dusk, thereby dampening *FT* mRNA expression upon its transcriptional activation by *CONSTANS*. This sets the correct level of *FT* expression at the end of long days, and thus prevents precocious flowering in response to the long-day cue.

## Introduction

The timing of the developmental transition from a vegetative to a reproductive phase is critical for reproductive success in flowering plants. Plants synchronize their timings of floral transition with changing seasons to flower at a suitable time. The change in day length or photoperiod is a key seasonal cue, especially at high latitudes, and is perceived in leaves, leading to the production of florigen in leaf vasculature [1],[2]. Florigen, a systemic signal, is transmitted through phloem from leaf to the shoot apical meristem (SAM), leading to flower formation [1]–[3]. Many plant species respond to day length changes through photoperiod pathways. According to their photoperiodic responses, plants are classified into long-day plants, i.e., flowering occurring only when the day becomes longer than a threshold length or accelerates when the day length increases, short-day plants whose flowering induced upon the day getting shorter, and day-neutral plants [4].

FLOWERING LOCUS T (FT), first identified in *Arabidopsis thaliana* [5],[6], and FT homologs in other species are the major component of the mobile florigen [7]–[10]. In *Arabidopsis*, a facultative long-day plant that flowers rapidly under inductive long days, the output of the photoperiod pathway *CONSTANS* (*CO*) activates *FT* expression in leaf veins [2],[11]. The circadian clock sets a high *CO* mRNA expression in the late afternoon in long days, which coincides with light exposure resulting in CO protein accumulation towards the day's end, because light stabilizes the CO protein [2],[4]. The vasculature-expressed CO protein promotes *FT* expression activation in the phloem companion cells specifically at the end of long days, resulting in rhythmic *FT* expression [2],[4]. During night, CO is rapidly degraded by proteasomes and *FT* expression is repressed. Upon its production in dusk, the FT protein moves from phloem to SAM to induce flowering. FT and its function as florigen are conserved in the flowering plants so far examined; the level of *FT* expression at the end of long days in *Arabidopsis* plays a primary determining role for when a plant to flower [1],[2]. To date, beside CO, other factors (if any) that directly regulate *FT* expression specifically at dusk remain to be identified.

Histone acetylation and de-acetylation can regulate chromatin structure and gene expression. Histone acetyltransferases (HATs) add acetyl groups to core histone tails and typically function to promote transcription of target loci, whereas histone deacetylases (HDACs) remove acetyl groups and are often linked with transcriptional repression [12]. Recent studies of genome-wide binding sites of mammalian HATs and HDACs reveal that both are targeted to actively transcribed genes to control their expression [13]. In addition, rapid and synchronous recruitment of HATs and HDACs to actively transcribed loci have been observed in yeast as well [14],[15]. At these loci HATs act typically to promote transcription, whereas HDACs deacetylate acetylated histones to maintain an adequate level of acetylation and/or reset the acetylation state of a target locus following transcription [13]–[15].

Histone acetylation is involved in plant gene regulation. In *Arabidopsis*, functional disruption of HATs such as GCN5 and AtHAC1, or HDACs including HDA6 and HDA19, leads to pleiotropic abnormalities in growth and development [16]–[19]. For instance, loss of *AtHAC1* or *HDA6* function causes upregulation of a potent floral repressor *FLOWERING LOCUS C* (*FLC*), resulting in a delay in flowering [17]–[19]. Certain HATs (e.g., GCN5) and HDACs (e.g., HDA6) are involved in genome-wide regulation of gene expression, as revealed by transcript profiling [16],[18], whereas others may control the expression of only a subset of loci. How a HAT or HDAC is recruited to its target loci remains elusive.

HDACs such as the reduced potassium dependency-3 (RPD3) type are often found in multiprotein co-repressor complexes, among which the yeast Sin3-HDAC complex has been well characterized [20],[21]. The core components of this complex typically include the master scaffold protein Sin3, the RPD3 HDAC, and several Sin3-associated structural components including Sin3-Associated Polypeptide 18 (SAP18) and Sin3-Associated Polypeptide 30 (SAP30); homologs of these components are also found in a mammalian Sin3-HDAC like complex [20]. This complex does not bind to DNA directly and is often recruited to specific loci through association with DNA-binding proteins [21]. In *Arabidopsis*, there are six *Sin3* homologs including *SNL1–SNL6* (*SNL* for *SIN3-LIKE*), and four close *RPD3* homologs including *HDA19*, *HDA9*, *HDA7*, and *HDA6* [22],[23]. In addition, there is only one *SAP18* homolog in *Arabidopsis*, *AtSAP18*, that has been shown to play a role in salt stress response and floral organ formation [24],[25]. The *Arabidopsis* genome does not encode an apparent homolog of the yeast SAP30; hence, whether there are Sin3-RPD3 like co-repressor complexes for histone deacetylation in *Arabidopsis* remains to be addressed.

Here we report that in *Arabidopsis* there are two functional relatives of the yeast SAP30, named as SAP30 FUNCTION-RELATED 1 (AFR1) and SAP30 FUNCTION-RELATED 2 (AFR2), acting as part of HDAC complexes (AFR1-HDAC or AFR2-HDAC) to modulate the acetylation level of *FT* chromatin upon *FT* activation at the end of long days (LDs) (16-h light/8-h dark). Both AFR1 and AFR2 proteins accumulate at the end of LDs, and bind to *FT* chromatin at dusk, but not in the middle of the day. Moreover, we found that the MADS-domain transcription factor AGAMOUS LIKE 18 (AGL18) directly interacts with AFR1 and AFR2 and recruits these proteins and presumably AFR1/AFR2-HDAC to *FT* chromatin specifically at the end of LDs leading to histone deacetylation upon *FT* activation. The output of the photoperiod pathway *CO* at the end of LDs not only activates *FT* expression, but also enables the recruitment of AFRs to the *FT* locus to dampen *FT* expression and set it at an adequate level, preventing precocious flowering in response to the day length cue in *Arabidopsis*.

## Results

### Two *Arabidopsis* Functional Relatives of the Yeast SAP30 (ScSAP30) Associate with Histone Deacetylases to Form HDAC Complexes

**Figure 1:**
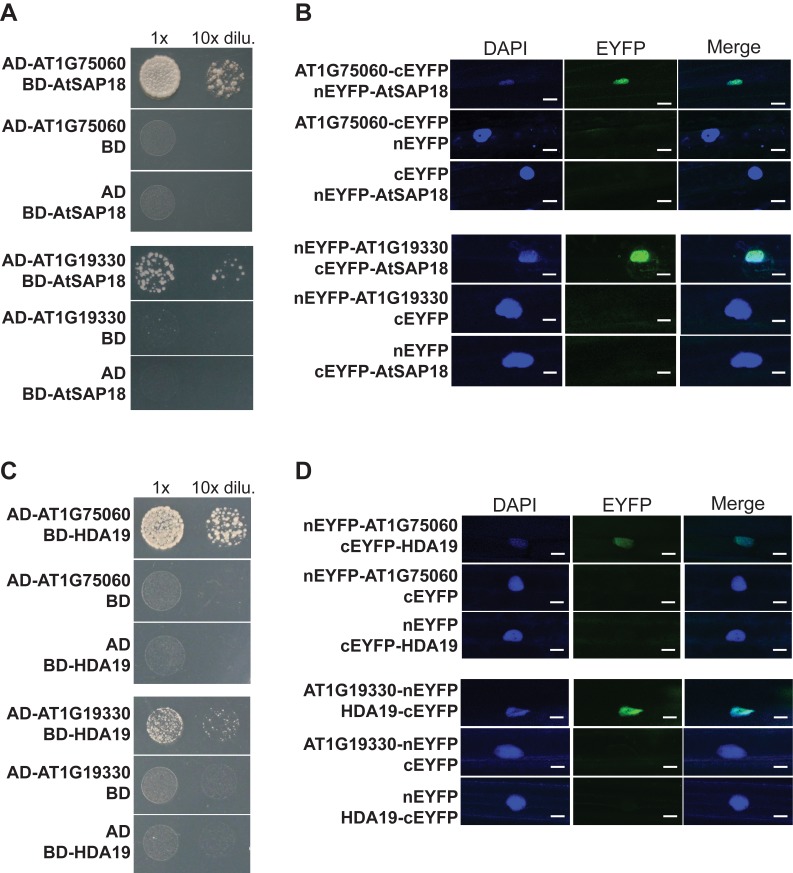
Direct interactions of AT1G75060 (AFR1) and AT1G19330 (AFR2) with AtSAP18 and HDA19 proteins. (A) Interactions of AtSAP18 with AT1G75060 and AT1G19330 in yeast. The indicated proteins of full-length were fused with the GAL4-BD or AD domain. Yeast cells harboring the fusion proteins, BD and/or AD (as indicated), were grown on selective synthetic defined media lacking of Trp, Leu, and His. (B) BiFC analysis of the interactions of AtSAP18 with AT1G75060 and AT1G19330 in onion epidermal cells. Onion epidermal cells were co-transformed transiently by a pair of plasmid, as indicated, via biolistic gene bombardment. Yellowish-green signals indicate physical associations of paired proteins in the nuclei. Blue fluorescence from a DAPI (4′,6-diamidino-2-phenylindole) staining indicates a nucleus. Bar = 20 µm. (C) Interactions of HDA19 with AT1G75060 and AT1G19330 in yeast. The indicated full-length proteins were fused with the GAL4-BD or AD domain. Yeast cells were grown on selective synthetic defined media lacking of Trp, Leu, and His. (D) BiFC analysis of the interactions of HDA19 with AT1G75060 and AT1G19330 in onion epidermal cells. Bar = 20 µm.

In an effort to explore whether a Sin3-SAP30-RPD3 co-repressor-like complex in *Arabidopsis* exists, we first examined whether *Arabidopsis* has any functional relatives of ScSAP30 because it lacks of an apparent homolog of the full-length ScSAP30. In the C-terminal region of ScSAP30 there is a domain important for its function: the 30-amino acid Sin3-binding region (SBR) [26]. We used C-SAP30 to BLAST the *Arabidopsis* protein database and found that there are nine proteins containing a region with a similarity to SBR, among which AT1G75060 and AT1G19330 are the top two hits (Figure S1A; unpublished data). We reasoned that if these two proteins are functional relatives of SAP30, they would directly associate with SNLs, AtSAP18, and/or RPD3-type HDACs. Yeast two-hybrid assays were performed to check direct interactions of AT1G75060 or AT1G19330 with SNL2 (SIN3-LIKE 2), AtSAP18, HDA9, and/or HDA19. Indeed, these proteins directly interacted with AT1G75060 and AT1G19330 (Figures 1A, 1C, and S2).

Next, to confirm these interactions in plant cells, we conducted bimolecular fluorescence complementation (BiFC) assays in which non-fluorescent N-terminal and C-terminal fragments of enhanced yellow fluorescent protein (EYFP) were fused to the full-length AT1G75060, AT1G19330, AtSAP18, or HDA19 proteins. When AT1G75060-cEYFP and nEYFP-AtSAP18 were simultaneously expressed in onion epidermal cells, in the nuclei fluorescence was observed (Figure 1B), demonstrating a direct interaction of AT1G75060 with AtSAP18. Similarly, we also examined and observed direct interactions of AT1G19330 with AtSAP18, AT1G19330 with HDA19, and AT1G75060 with HDA19 in the nuclei of onion cells (Figure 1B and 1D). These results suggest that both AT1G75060 and AT1G19330 function like ScSAP30, namely, as an integral component of Sin3-RPD3 co-repressor like complexes, although there is little similarity of amino acid sequence between these proteins and ScSAP30 outside of the C-terminal SBR (Figure S1A). Hence, the homologous AT1G75060 and AT1G19330 proteins were named as AFR1 and AFR2, respectively. In short, our findings collectively suggest that RPD3-type HDACs and AFRs together with other structural components such as SNLs and AtSAP18 form Sin3-SAP30-SAP18-RPD3 like complexes in *Arabidopsis*.

### *AFR1* Acts Additively with *AFR2* to Repress the Floral Transition

**Figure 2:**
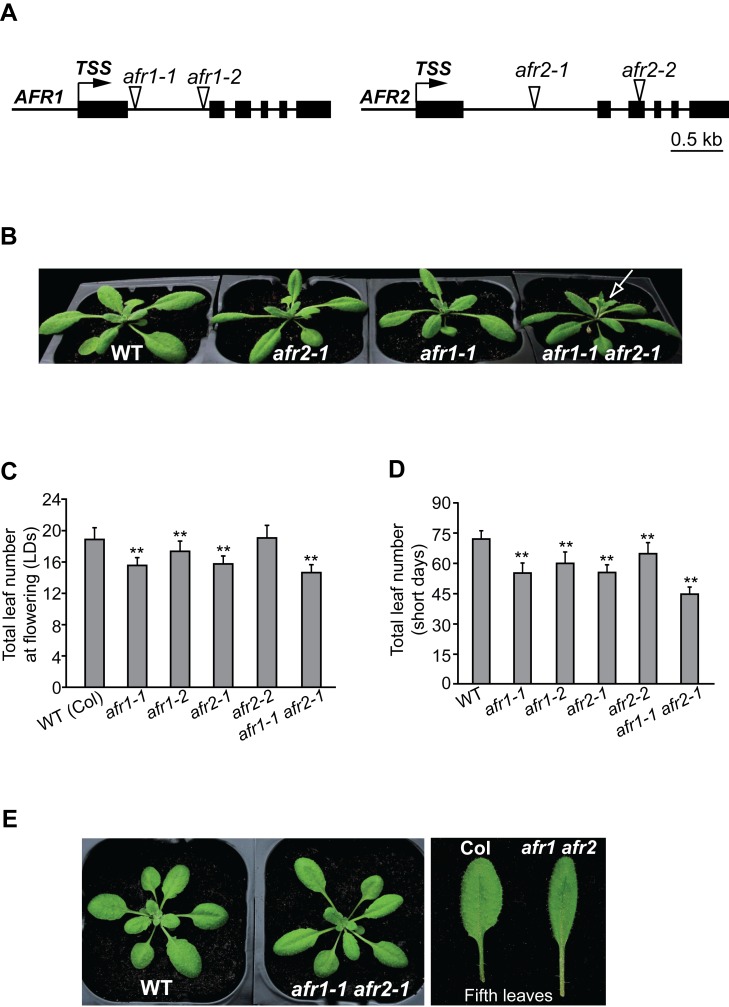
Phenotypes of *afr1*, *afr2*, and *afr1 afr2* mutants. (A) *AFR1* and *AFR2* gene structures. Exons are represented by black boxes, and arrows indicate transcription start sites (TSS); triangles for *T-DNA* insertion sites. (B) *afr1*, *afr2*, and *afr1 afr2* mutants grown in LDs. The arrow indicates a main bolt with flowers. (C) Flowering times of the indicated genotypes grown in LDs. 19–23 plants were scored for each line. Double asterisks indicate statistically significant differences in the means between Col (WT) and the indicated mutants, as revealed by two-tailed Student's *t* test (**, *p*<0.01). Bars indicate SD (for standard deviation). (D) Flowering times of the indicated genotypes grown in short days. 11–15 plants were scored for each line. Double asterisks indicate statistically significant differences in the means between Col and the indicated mutants. (E) Leaf phenotype of the *afr1 afr2* double mutant grown in LDs.

To elucidate the biological functions of *AFR1* and *AFR2*, we identified their loss-of-function mutants, and each carries a transfer DNA (*T-DNA*) insertion. In *afr1-1* and *afr1-2*, a *T-DNA* is inserted into the first intron of *AFR1*, resulting in a great reduction, but not elimination of *AFR1* expression (Figures 2A and S3A). In both *afr2* alleles, full-length *AFR2* transcripts were not detected, but *AFR2* transcripts truncated at their 3′ ends were detectable in the *afr2-2* mutant, which are predicted to encode a protein containing the first 149 amino acids of AFR2 (Figure S3B). Grown in LDs, *afr1-1*, *afr1-2*, and *afr2-1* mutants flowered moderately earlier than the wild type (WT) Col, as revealed by the total number of leaves formed prior to flower formation from the primary SAM of an examined line, the developmental standard for *Arabidopsis* flowering-time measurement (Figure 2B and 2C). In short days (8-h light/16-h dark), all *afr1* and *afr2* mutants flowered earlier than WT (Figure 2D), and the early-flowering phenotypes of *afr1-1* and *afr2-1* were fully rescued by complementation with tagged *AFR1* and *AFR2*, respectively (Figure S4). Of note, *afr2-2* is a weak allele compared to *afr1-1*, and it is likely that the truncated AFR2 may retain some function in the *afr2-2* mutant. We further constructed the *afr1 afr2* double mutant (*afr1-1 afr2-1*), and found it flowered earlier than either single mutant in both LDs and short days (Figures 2C, 2D, and S5A). Taken together, these results suggest that *AFR1* and *AFR2* act additively to repress the floral transition in *Arabidopsis*, indicating that the homologous AFR1 and AFR2 proteins do not function in one complex. In addition to early flowering, the *afr1 afr2* mutant exhibited a leaf phenotype: longer petiole, narrower leaf blade, and with a slightly increased leaf initiation rate (Figures 2E and S5C), suggesting that *AFR1* and *AFR2* may also be required for proper leaf development.

### *AFR1* and *AFR2* Downregulate *FT* Expression Specifically at the Day's End to Delay Flowering in LDs

**Figure 3:**
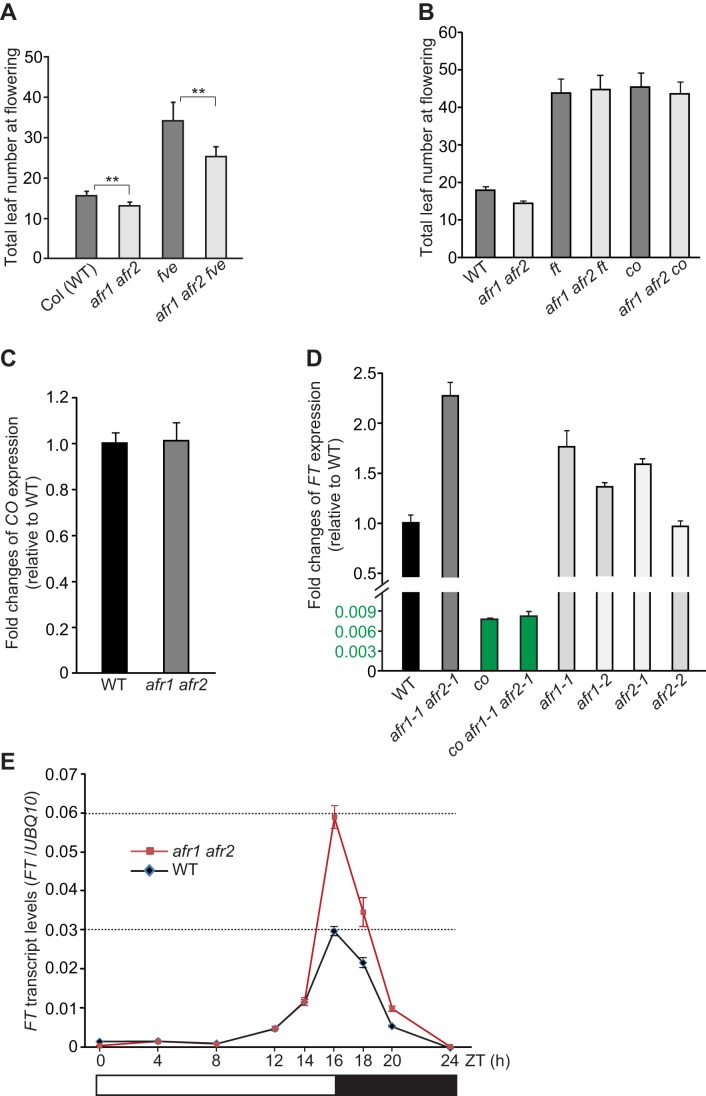
*AFR1* acts additively with *AFR2* to downregulate *FT* expression specifically at the day's end in LDs. (A,B) Flowering times of the indicated genotypes grown in LDs. 11–17 plants were scored for each line. The *afr1 afr2* double mutant is *afr1-1 afr2-1*. Double asterisks indicate statistically significant differences in the means between the indicated genotypes, as revealed by two-tailed Student's *t* test (**, *p*<0.01). Bars indicate SD. (C,D) Relative *CO* (C) and *FT* transcript levels (D) in the seedlings of indicated genotypes at the end of LDs (ZT16), quantified by qRT-PCR. The transcript levels were normalized first to the endogenous control *UBQ10*, and relative fold changes to WT are presented (note that the *FT* transcript level in *co* was less than 1% of that in WT). Bars indicate SD of triplicate measurements. One of two biological repeats with similar results is shown. (E) *FT* mRNA levels in Col and *afr1 afr2* seedlings over a 24-h LD cycle, as quantified by qRT-PCR. *FT* transcript levels were normalized to *UBQ10*; bars indicate SD of triplicate measurements. A biological repeat of this analysis is included as Figure S8A. White and dark bars below the x-axis indicate light and dark periods, respectively.

There are several genetic pathways promoting flowering in *Arabidopsis*, including the autonomous, photoperiod, vernalization, and thermosensory pathways [2],[27],[28]. To explore in which genetic pathway *AFR1* and *AFR2* act to regulate flowering, we introduced the photoperiod-pathway mutation *co* or a null mutant of *FVE*, a component of the autonomous and thermosensory pathways, into the *afr1 afr2* double mutant [27],[28]. The late-flowering phenotype of *fve* was partly suppressed by *afr1 afr2*, whereas the *afr1 afr2 co* triple mutant flowered at about the same time as *co* (Figure 3A and 3B); thus, the *co* mutation is epistatic to *afr1* and *afr2*. Furthermore, we found that *ft*, like *co*, is epistatic to *afr1* and *afr2* (Figure 3B). These results suggest that *AFR1* and *AFR2* act through the *CO-FT* regulatory module of the photoperiod pathway to control flowering.

Next, we examined *CO* expression upon loss of *AFR1* and *AFR2* function at the end of LDs (ZT16, 16 h after light on or dawn; Zeitgeber time [ZT]), because *CO* is highly expressed to activate *FT* expression at this time point. *CO* expression remained unchanged in the *afr1 afr2* double mutant compared to WT (Figure 3C); additionally, loss of *CO* function didn't affect *AFR1* or *AFR2* expression (Figure S6). We further examined *FT* expression in *afr1*, *afr2*, and *afr1 afr2* seedlings at the end of LDs. *FT* transcript levels were increased moderately in *afr1* and *afr2* single mutants and in the double mutant (Figure 3D), consistent with that *AFR1* and *AFR2* act additively to repress flowering. In addition, *FT* expression was slightly upregulated in *afr1 afr2* in short days, though its transcript levels were very low in both WT and *afr1 afr2* plants (Figure S7). Taken together, these results show that *AFR1* and *AFR2* function additively to downregulate *FT* expression and so delay flowering. We further measured *FT* transcript levels in *co* and *afr1 afr2 co* seedlings at ZT16, and found that *co* completely suppressed *FT* up-regulation in the *afr1 afr2* background (Figure 3D). Hence, *CO* is required for *FT* downregulation at ZT16 by *AFR1* and *AFR2*.

*FT* expression is activated by *CO* specifically at the end of LDs, and repressed at other times of the day, resulting in rhythmic *FT* mRNA expression at dusk in LDs. Several histone modifiers including the histone H3 lysine-27 (H3K27) methytransferase CLF and the PKDM7B H3 lysine-4 demethylase (also known as JMJ14 or AtJMJ4), are required for continuous *FT* repression along day/night cycles [29]–[32]. We explored whether *AFR1* and *AFR2* could repress *FT* expression in other times of LDs beside ZT16. Upon loss of *AFR1* and *AFR2* function, *FT* expression in LDs was not affected from ZT0 through ZT14, but upregulated only from ZT14 to ZT20 (Figures 3E and S8A). Thus, in LDs *AFR1* and *AFR2* downregulate *FT* expression specifically in the presence of CO activity at the day's end, to delay flowering.

### *AFR1* and *AFR2* Downregulate *FT* Expression in Leaf Vasculature

**Figure 4:**
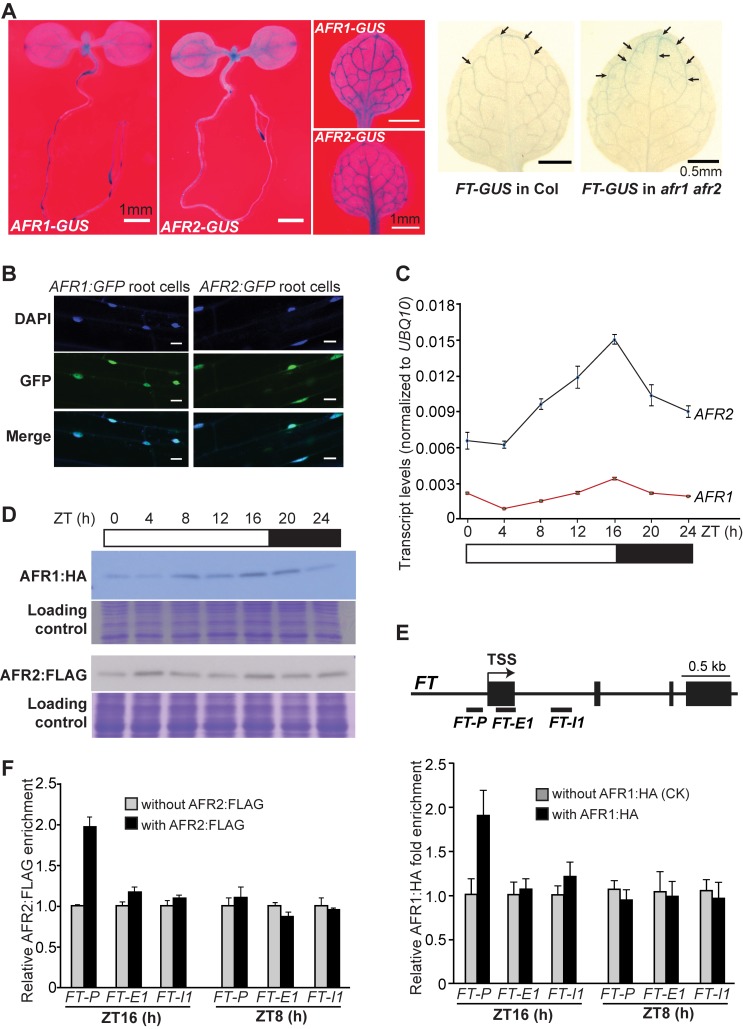
Analyses of AFR1 and AFR2 expression patterns and their bindings to *FT* chromatin. (A) Spatial expression patterns of *AFR1-GUS*, *AFR2-GUS*, and *FT-GUS*. LD-grown Col seedlings or rosette leaves were stained for 6 h except for *AFR1-GUS* staining with 8.5 h. Arrows indicate stained veins. (B) Nuclear localization of the AFR1:GFP and AFR2:GFP fusion proteins in *Arabidopsis* root cells. Scale bars are 50 µm. The blue DAPI staining indicates nuclei. (C) *AFR1* and *AFR2* mRNA levels in Col (WT) seedlings over a 24-h LD cycle. The mRNA levels were normalized to *UBQ10*; bars indicate SD of triplicate measurements. A biological repeat of this analysis is included as Figure S8B. White and dark bars below the x-axis indicate light and dark periods, respectively. (D) AFR1:HA and AFR2:FLAG protein levels in Col seedlings over a 24-h LD cycle. Total proteins loaded in a duplicated SDS-PAGE gel were stained with Coomassie Blue, serving as loading controls. (E) ChIP analysis of AFR1:HA enrichment at the *FT* locus. Amounts of the immunoprecipitated genomic fragments were measured by qPCR, and normalized first to the endogenous control *TUBULIN8* (*TUB8*). The fold enrichment of AFR1:HA in each examined region (at each time point) was calculated by dividing the *TUB8*-normalized amount of examined region from the AFR1:HA-expressing line, by that of WT (without *AFR1:HA*) at each time point. Error bars indicate SD of triplicate quantifications (technical replicates). A biological repeat of this analysis is presented as Figure S10A. (F) ChIP analysis of AFR2:FLAG enrichment at the *FT* locus. The fold enrichments of AFR2:FLAG were calculated in a way similar to those of AFR1:HA. Error bars indicate SD of triplicate quantifications. A biological repeat of this analysis is presented as Figure S10B.

Day length is perceived in leaves, leading to *FT* activation specifically in the phloem companion cells of leaf veins [1],[2]. We asked whether *AFR1* and *AFR2* are expressed in leaf veins to downregulate *FT* expression. To this end, genomic *AFR1* and *AFR2* fragments (including 5′ promoter plus part of the coding region of each gene), were fused to the *β-GLUCURONIDASE* (*GUS*) gene in frame and the fusions were introduced into *Arabidopsis* by transformation. Histochemical staining revealed that both *AFR1* and *AFR2* were strongly expressed in leaf vasculature in addition to root tips and shoot apices (Figure 4A). To examine in which tissues *AFR1* and *AFR2* downregulate *FT* expression, we introduced *afr1-1* and *afr2-1* into an *FT-GUS* reporter line [33], and found that the *GUS* expression directed by a *FT* promoter was upregulated only in leaf veins (Figure 4A). Thus, *AFR1* and *AFR2* downregulate *FT* expression in the leaf vasculature. Next, we examined the subcellular localization of both AFR1 and AFR2 proteins by fusing them with green fluorescence protein (GFP). Upon their expression in *Arabidopsis* roots, these fusion proteins were specifically localized in the nuclei (Figure 4B), consistent with that AFR1 and AFR2 act as part of nuclear AFR1/AFR2-HDAC complexes in leaf veins to downregulate *FT* expression.

### AFR1 and AFR2 Proteins Accumulate and Directly Interact with the *FT* locus at the End of LDs

We hypothesized that the downregulation of *FT* expression by *AFR1* and *AFR2* in the presence of CO activity at the end of LDs may be attributed to AFR-HDAC binding to *FT* chromatin only at this time in LDs. To test this hypothesis, we first examined the diurnal expression patterns of *AFR1* and *AFR2* in LDs, and found that the expression of both genes peaked at the day's end (Figure 4C). Next, using the functional AFR1:HA-expressing line and the functional AFR2:FLAG-expressing line (driven by their native promoters; see Figure S4), we measured the abundance of both proteins every 4 h over a 24-h LD cycle. The levels of AFR1 and AFR2 proteins changed diurnally in LDs, and both proteins accumulated at the end of the day (Figures 4D and S9). This indicates that AFR1/AFR2-HDAC may accumulate at the end of LDs.

We further explored whether AFR1 could bind to *FT* chromatin, and if so, at what time of the day. Chromatin immunoprecipitation (ChIP) assays were performed using the AFR1:HA-expressing seedlings. We found that at the end of LDs (ZT16), AFR1 bound to the proximal *FT* promoter region, but not in the first exon or the first intron (Figures 4E and S10A). Unlike at ZT16, AFR1 was not enriched at all in the *FT* locus in the middle of the day (ZT8) (Figure 4E). Moreover, using ChIP with the AFR2:FLAG-expressing seedlings, we uncovered that AFR2, like AFR1, bound to the proximal *FT* promoter region at ZT16, but not at ZT8 (Figures 4F and S10B). The bindings of AFR1 and AFR2 to *FT* chromatin at the end of LDs coincide well with that both proteins accumulate at this time. Taken together, these results suggest that in LDs AFR1/AFR2-HDAC binds to *FT* chromatin specifically at the day's end to modulate *FT* expression in the presence of CO activity.

### AFR1 and AFR2 Mediate Periodic Histone Deacetylation at *FT* at the End of LDs

**Figure 5:**
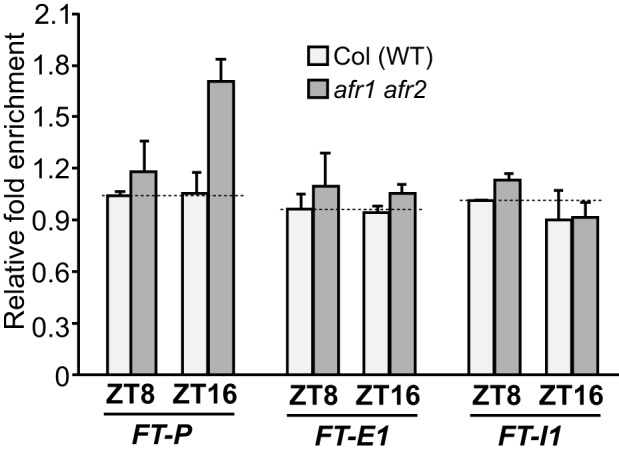
ChIP analysis of levels of acetylated histone H3 in Col and *afr1 afr2* rosette leaves. Amounts of the immunoprecipitated genomic fragments were quantified by qPCR. The fold enrichments were calculated as follows: for each examined *FT* region, the amount of DNA fragments from WT or *afr1 afr2* at each time point (ZT8 or ZT16) was first normalized to the constitutively expressed *TUBULIN2* (*TUB2*) in each sample, and subsequently, the *TUB2*-normalized values for the *afr1 afr2* at ZT8, the *afr1 afr2* at ZT16, or the WT at ZT16 were divided by the value for the WT at ZT8 to obtain fold enrichments. Shown are the means and SD of two ChIP experiments. An analysis of H3 acetylation on *FT* chromatin in Col and *afr1 afr2* seedlings is presented in Figure S11.

The recruitment of AFR1 and AFR2 to *FT* chromatin at the day's end, may cause periodic histone deacetylation in LDs. To test this, we examined steady-state levels of histone H3 acetylation of *FT* chromatin in WT and *afr1 afr2* seedlings (and rosette leaves) at ZT8 and ZT16. Loss of *AFR1* and *AFR2* function led to an increase of steady-state level of acetylated H3 in the proximal *FT* promoter region, but not in the first exon or the first intron at ZT16, whereas at ZT8 there was no noticeable change in all three regions examined (Figures 5 and S11). This is consistent with the binding of AFR1 and AFR2 to *FT* chromatin in the proximal promoter at the end, but not the middle of LDs (Figure 4E). Together, these results show that AFR1 and AFR2 (presumably as part of AFR1/AFR2-HDAC) mediate histone deacetylation at the *FT* locus to downregulate its expression at the end of LDs. Interestingly, there is no apparent increase of the steady-state acetylation levels of *FT* chromatin in WT at ZT16 relative to ZT8, although *FT* expression is activated at ZT16 (Figure 5; note that the relative acetylation level of each sample was normalized to the WT at ZT8). Therefore, there must be active histone acetylation by HAT(s) on *FT* chromatin to “counteract” the AFR-mediated histone deacetylation at the end of LDs.

### The Transcription Factor AGL18 recruits AFRs to the *FT* Locus Specifically at the End of LDs

**Figure 6:**
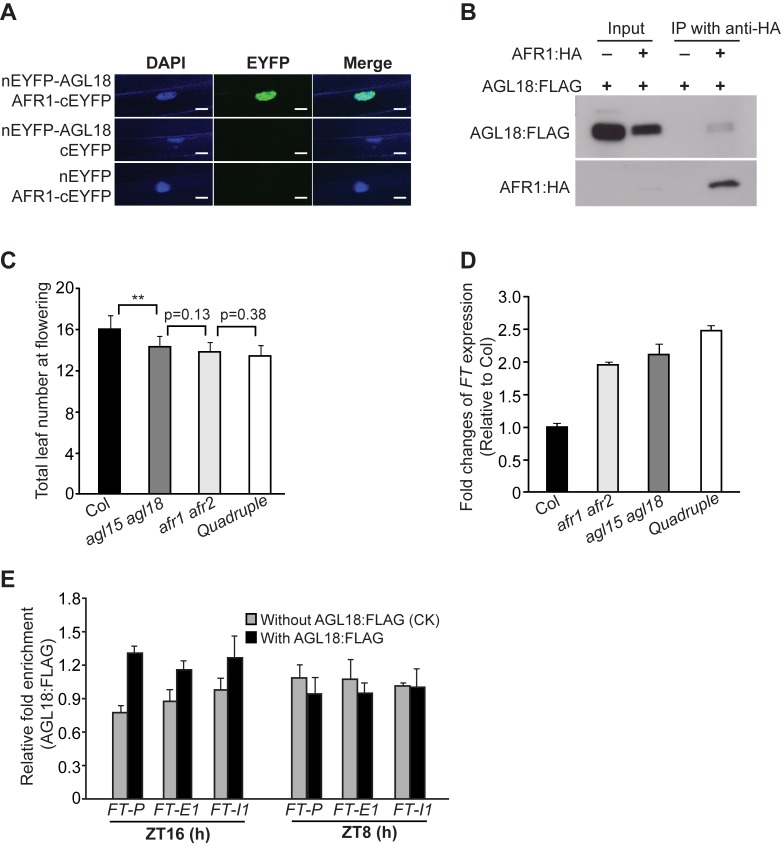
AGL18 directly interacts with AFR1 and binds to *FT* chromatin at the day's end in LDs. (A) BiFC analysis of the interaction of AGL18 with AFR1 in onion epidermal cells. Yellowish-green signals indicate the physical association of AGL18 with AFR1 in the nuclei (indicated by the blue fluorescence from DAPI). Bar = 20 µm. (B) Co-immunoprecipitation of AFR1 with AGL18 in *Arabidopsis* seedlings. Total protein extracts from F_1_ seedlings of the doubly hemizygous *AGL18:FLAG* and *AFR1:HA*, were immunoprecipitated with anti-HA agarose; subsequently, the precipitates were analyzed by western blotting with anti-FLAG (recognizing AGL18:FLAG) and anti-HA (recognizing AFR1:HA). (C) Flowering times of the indicated genotypes grown in LDs. 12–16 plants were scored for each line. Double asterisks indicate a statistically significant difference in the means between Col and *agl15 agl18*, as revealed by two-tailed Student's *t* test (**, *p*<0.01). Bars indicate SD. (D) Relative *FT* transcript levels in the seedlings of indicated genotypes at ZT16, quantified by qRT-PCR. The transcript levels were first normalized to *UBQ10*, and relative fold changes to Col are presented. Bars indicate SD of triplicate measurements. One of two biological repeats with similar results is shown. (E) ChIP analysis of AGL18:FLAG enrichment at the *FT* locus. Amounts of immunoprecipitated genomic fragments were measured by qPCR, and normalized first to the endogenous control *TUB8*. The fold enrichment of AGL18:FLAG in each examined region (at each time point) was calculated by dividing the *TUB8*-normalized amount of examined region from the AGL18:FLAG-expressing line, by that of WT (without *AGL18:FLAG*) at each time point. Error bars indicate SD of triplicate measurements. A biological repeat of this analysis is presented as Figure S13B.

In yeast SAP30 is involved in the recruitment of SAP30-Sin3-RPD3 co-repressor complex to a target locus [21]. We hypothesized that transcription factors may directly interact with AFR1 and AFR2 to recruit AFR1/AFR2-HDAC to the *FT* locus at the end of LDs. Previous studies have revealed that several MADS-domain transcription factors including FLC, SHORT VEGETATIVE PHASE (SVP), AGL18, and AGAMOUS LIKE 15 (AGL15), are involved in *FT* repression in the vasculature to inhibit flowering [28],[34],[35]. Using a candidate gene approach, we found that the AGL18 protein directly interacted with AFR1 and AFR2 in the nuclei of onion epidermal cells, as revealed by the BiFC experiments (Figures 6A and S12A). AGL18, like AFR1 and AFR2, are also expressed in leaf veins [35]. To further confirm the *in vivo* association of AGL18 with AFR1, we conducted co-immunoprecipitation experiments using the seedlings expressing AFR1:HA and a functional AGL18:FLAG (Figure S13A), and found that anti-HA (recognizing AFR1:HA) immunoprecipitated AGL18:FLAG from the seedlings (Figure 6B). Thus, indeed, AGL18 directly interacts with AFR1 and presumably AFR1-HDAC in *Arabidopsis*.

*AGL18* acts redundantly with its homolog *AGL15* to repress flowering [35]. We further found that AFR1 directly interacted with AGL15 in yeast and plant cells (Figure S12B and S12C). Of note, it has been previously shown that AGL15 could interact moderately with the conserved HDAC domain of HDA19 in yeast cells [36], which can directly associate with AFR1 and AFR2 (Figure 1C and 1D). Next, we examined the genetic interaction of *afr1 afr2* with *agl15 agl18* in flowering-time regulation. Grown in LDs, the *afr1 afr2 agl15 agl18* quadruple mutant flowered at a similar time to either double mutant (Figure 6C), suggesting that these four genes act in the same flowering-regulatory pathway. Further *FT* expression analysis revealed that *FT* transcript levels (at ZT16) in both *afr1 afr2* and *agl15 agl18* seedlings increased to about 2-fold of the WT level, and that in the quadruple, *FT* levels were slightly higher than either double mutant (Figure 6D). This slight increase is not surprising given that neither double mutant may not be null as there are low levels of full-length *AFR1*, and *AGL15* and *AGL18* transcripts in *afr1* and *agl15 agl18*, respectively (Figures S3 and S14). Taken together, these data are in line with that the DNA-binding AGL15 and AGL18 may act to recruit AFR1 and AFR2 to downregulate *FT* expression.

**Figure 7:**
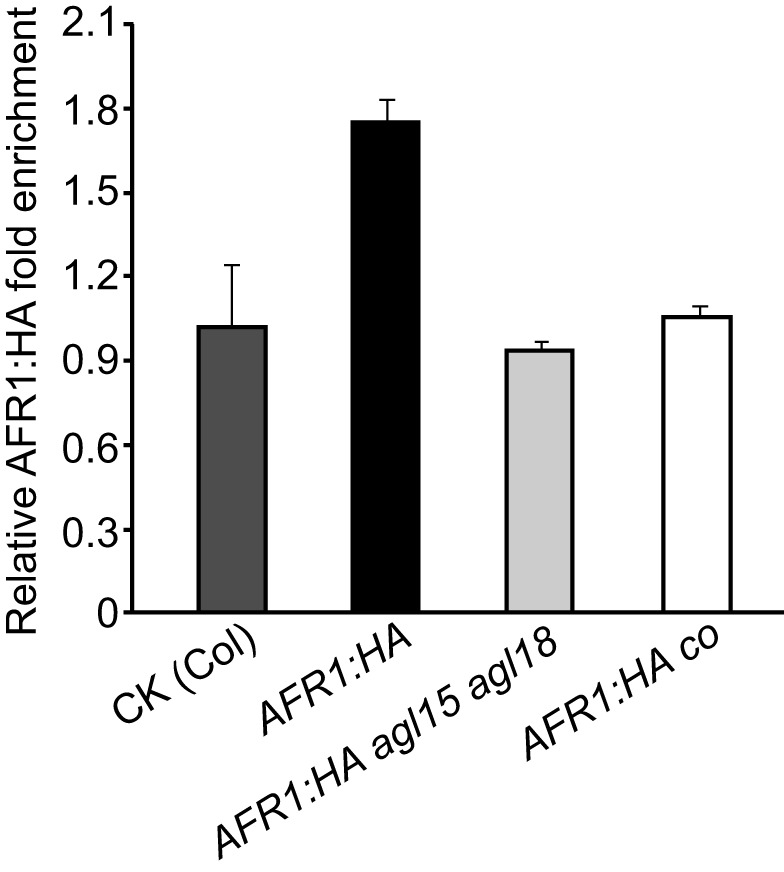
AFR1 binding to *FT* chromatin at the end of LDs requires *CO*, *AGL15*, and *AGL18*. Seedlings of *afr1 AFR1:HA*, *agl15 agl18 afr1 AFR1:HA*, *co afr1 AFR1:HA*, and WT (negative CK) were harvested at ZT16 and subjected to ChIP assays with anti-HA. The fold enrichments of AFR1:HA in *FT-P* (a proximal promoter region) in the AFR1:HA-expressing lines over CK, are presented. Error bars indicate SD of triplicate measurements. A biological repeat of this analysis is presented as Figure S10C.

To explore the role of AGL18 for AFR1 recruitment, first we examined whether and when AGL18 could directly interact with the *FT* locus. Using ChIP with the AGL18:FLAG-expressing seedlings, we found that at the end of LDs AGL18:FLAG, like AFR1, was apparently enriched in the proximal *FT* promoter region, in addition to a slight enrichment in the first exon (Figure 6E). In the middle of LDs, AGL18 was not associated with *FT* chromatin (Figure 6E). Thus, AGL18 directly interacts with the *FT* locus specifically at the end of LDs. Next, we investigated whether AGL18 is required for AFR1 recruitment to *FT* chromatin at the end of LDs. The line expressing the functional AFR1:HA (in *afr1-1* background) was crossed to *agl15 agl18*, and the seedlings of *afr1 AFR1:HA* and *agl15 agl18 afr1 AFR1:HA* harvested at ZT16 were subjected to ChIP assays with anti-HA. We found that loss of *AGL15* and *AGL18* function nearly eliminated AFR1 binding to *FT* chromatin at the end of LDs (Figure 7). These results, together with the direct interactions of AGL18 and AGL15 with AFR1 and AFR2, led us to infer that these two MADS-domain transcription factors recruit AFRs (presumably AFR-HDAC) to the *FT* locus specifically at the end of LDs to dampen *FT* expression in the presence of CO activity.

### CO Activity Is Required for the AFR1 Recruitment to *FT* Chromatin at the End of LDs

As we showed earlier, *CO* is required for *AFR1*- and *AFR2*-mediated *FT* downregulation at the end of LDs. We reasoned that the CO activity at the *FT* locus at the end of LDs may be required for AGL18/AGL15 recruiting AFRs to *FT*. To test this, first we crossed the AFR1:HA-expressing line (in *afr1-1*) to *co* and created *co afr1 AFR1:HA*. Next, we carried out ChIP assays with the *AFR1:HA*-expressing seedlings harvested at ZT16, and found that *CO*, indeed, is required for AFR1 binding to *FT* chromatin at ZT16 (Figure 7). Upon its accumulation towards dusk in LDs, CO directly binds to the *FT* proximal promoter to promote its expression [37]. This raises a possibility that CO might directly interact with AGL18 and/or AFRs to recruit these proteins to the *FT* proximal promoter. Using the yeast-two-hybrid approach we found that CO didn't directly interact with AGL18, AFR1, or AFR2 (Figure S15); hence, we conclude that the CO protein is not directly involved in the recruitment of AFRs to *FT* chromatin. Taken together, these results suggest that the CO-mediated transcriptional activation of *FT* at the end of LDs enables or gates the recruitment of AFR-HDAC by AGL18/AGL15 to the *FT* locus, leading to the dampening of *FT* expression and so preventing precocious flowering in response to the day-length cue.

## Discussion

In this study, we have revealed that the plant-unique AFR1 and AFR2 mediate periodic histone deacetylation on the rhythmically expressed florigen gene *FT* to dampen its expression specifically at dusk in LDs in *Arabidopsis*. The output of the photoperiod pathway *CO* at the end of LDs not only activates *FT* expression, but also enables the recruitment of AFR-HDAC to the *FT* locus to dampen *FT* expression and set it at an adequate level, conferring a proper flowering time in response to the day length cue. The MADS-domain transcription factor AGL18 directly interacts with the AFR1 and AFR2 proteins that accumulate at dusk, and recruits AFR1 (presumably AFR1/AFR2-HDAC) to the *FT* locus at the end of LDs to modulate *FT* expression. Our findings collectively uncover a temporal chromatin mechanism, namely periodic histone deacetylation, for the day-length regulation of flowering time in higher plants.

### Dynamic Cycles of Histone Acetylation and Deacetylation at the *FT* Locus

The histone acetylation mark added by HATs can be rapidly removed by HDACs. Both HATs and HDACs have been found to associate with actively transcribed genes in yeast and human, and are often synchronously recruited to certain target loci, leading to dynamic cycles of acetylation and deacetylation [13]–[15]. HATs can bind transiently to and add acetyl groups to certain loci where HDACs transiently bind to erase the acetylation mark, resulting in constant acetylation and de-acetylation cycles at these loci [13]–[15]. Our study reveals that at the *FT* locus, accompanying rhythmic CO-mediated *FT* activation at the end of LDs, AFR1 and AFR2 (presumably AFR-HDAC) are recruited to the actively transcribed *FT* chromatin for histone deacetylation. Given that disruption of *AFR1* and *AFR2* function leads to an increase of steady-state level of acetylation on *FT* chromatin at the end of LDs, there must be a HAT (or HATs) to add acetyl groups, suggesting that constant cycles of acetylation and de-acetylation occur at the *FT* locus upon its activation by *CO* at dusk. AFR1/AFR2-HDAC is expected to act antagonistically to a HAT to regulate *FT* expression. Of note, the HAT(s) has not been identified yet.

The dynamic histone acetylation and deacetylation cycles in the proximal *FT* promoter may result from transient binding of HATs and AFR1/AFR2-HDAC to *FT* chromatin. Consistent with the transient or unstable AFR1-HDAC binding, our ChIP analysis shows that AFR1 is moderately enriched at the *FT* promoter (Figures 4E and 7). The increase of steady-state acetylation level at *FT* and *FT* upregulation upon disruption of AFR1/AFR2 function, indicate that at the end of LDs histone acetylation promotes *FT* transcription, whereas histone deacetylation executed by AFR-HDAC acts to downregulate *FT* expression. It is very likely that when *FT* chromatin is actively transcribed upon transcriptional activation by *CO* at the end of LDs, AFR-HDAC may remove acetyl groups following each cycle of HAT activities associated with *FT* transcription to reset the acetylation state of *FT* chromatin, and functions to dampen *FT* expression at dusk. The AFR-HDAC activities at dusk may also serve to reset *FT* chromatin rapidly to a silent state with a low level of acetylation at night. Consistent with this notion, we have observed that upon loss of *AFR1* and *AFR2* function, it takes several hours longer to reset *FT* expression to a silent state at night (Figures 3E and S8A).

Interestingly, the steady-state acetylation levels of *FT* chromatin, as measured by ChIP, remain largely unchanged from the middle to the end of LDs (Figure 5), although *FT* expression is switched on at dusk. Not surprisingly, this may reflect the dynamic cycles of histone acetylation and deacetylation upon *FT* expression induction by CO. In the budding yeast, during transcriptional induction of *ARG1* (encoding an argininosuccinate synthase), the Gcn5 HAT and several HDACs are synchronously recruited to *ARG1* chromatin, and these opposing activities result in no noticeable changes of steady-state acetylation levels at the *ARG1* locus, as measured by ChIP [14]. A similar situation has been observed during the transcriptional induction of several heat-responsive genes by heat shock in yeast [15].

### Role of CO in Gating the Recruitment of AFR1/AFR2-HDAC to *FT* Chromatin at the End of LDs

In this study we have revealed that the MADS-domain transcription factor AGL18 and presumably AGL15 act to recruit AFR1/AFR2-HDAC to the *FT* locus at the end of LDs. *AGL18* is well expressed from ZT8 to ZT16 (unpublished data), but the AGL18 protein binds to *FT* chromatin (in the proximal promoter) only at ZT16. This indicates that the chromatin state of the *FT* locus may play a role for the AGL18 binding to it; for instance, the state may determine whether a *cis*-element such as a CArG motif, recognized typically by a MADS domain, in *FT* promoter is accessible to AGL18.

We found that the output of the photoperiod pathway *CO* is required for, but not directly involved in the recruitment of AFR1 to *FT* chromatin at dusk. The CO protein directly binds to *FT* proximal promoter and also associates with NY-F transcriptional factors that bind to *FT* distal promoter, to promote *FT* expression specifically at the end of LDs [37]–[40]. Given the indirect role of CO for AFR1/AFR2 recruitment, we infer that at the end of LDs the binding of CO to the *FT* proximal promoter and/or consequent transcriptional activation of *FT* expression enable AGL18 recruiting AFRs to the *FT* locus. It is likely that CO activity may cause a change in *FT* chromatin state so that the *cis*-element(s) such as a CArG motif or its variant in the *FT* promoter become accessible by AGL18. In short, in the photoperiodic flowering regulation *CO* plays a dual role: activating *FT* expression and enabling AFR-HDAC-mediated *FT* downregulation to set a proper level of *FT* expression at the end of LDs.

**Figure 8:**
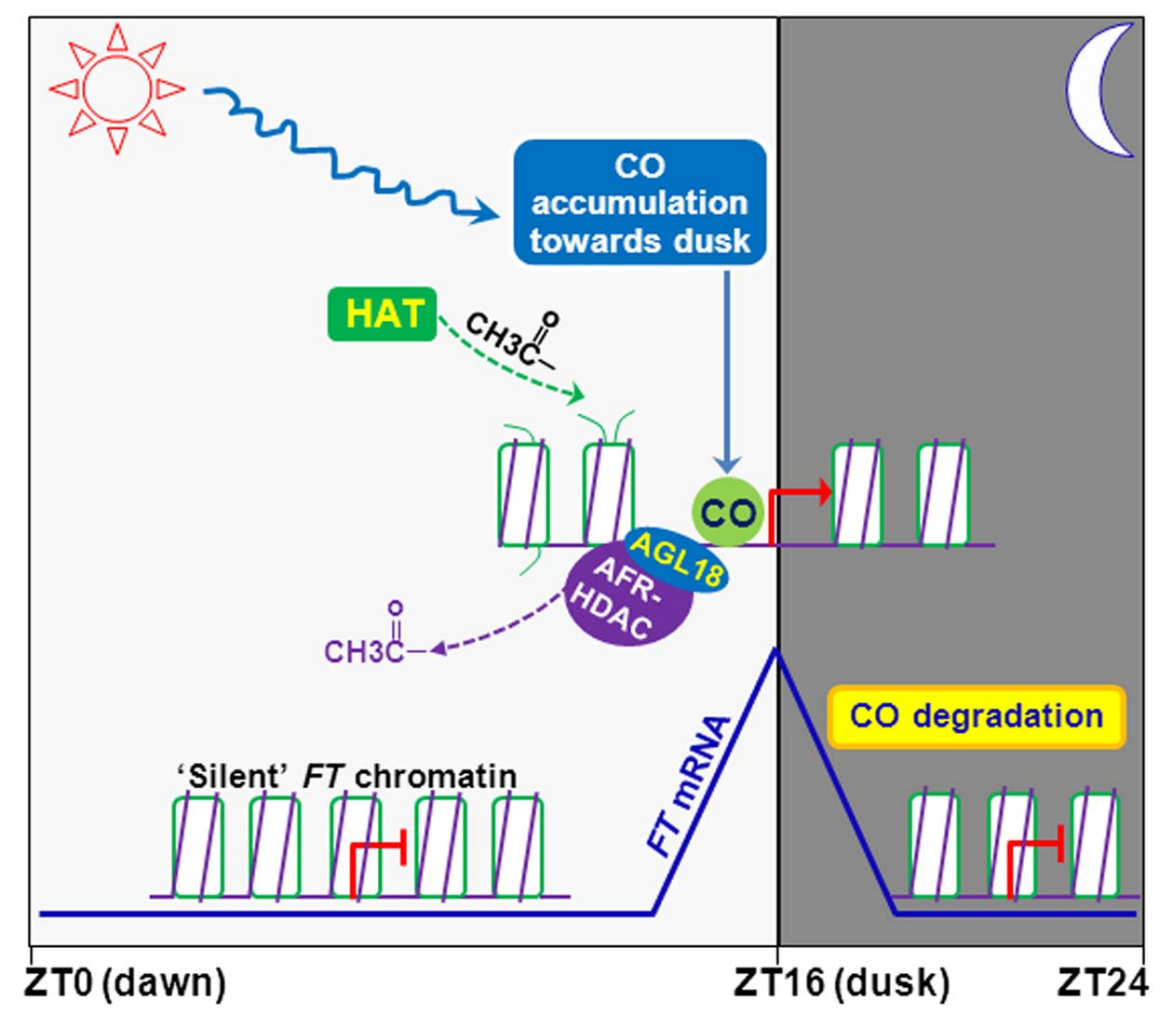
A working model for control of *FT* expression by the dynamic cycles of histone acetylation and deacetylation at the end of LDs. The coincidence of high *CO* mRNA expression with light exposure at the day's end leads to the CO protein accumulation towards dusk. CO directly binds to the *FT* proximal promoter, and CO activity at the *FT* locus may change the chromatin state and enables/gates AGL18 (and presumably AGL15) binding to the *FT* proximal promoter. AGL18 recruits AFR1/AFR2-HDAC to *FT* chromatin at dusk. In addition, the CO activity may also enable the recruitment of a HAT to *FT* chromatin. The opposing activities of HAT and AFR-HDAC on *FT* chromatin at the end of LDs conceivably modulate the acetylation dynamics of *FT* chromatin and set *FT* expression at an adequate level at dusk. At night, CO is rapidly degraded by proteasomes, which prevents the actions of HAT and AFR-HDAC on *FT* chromatin, resulting in a “silent” chromatin state. In early day, *FT* chromatin remains ‘silent’ due to lack of the CO protein. Day and night are indicated with white and gray shadings, respectively.

The accumulation of both AFR1 and AFR2 at the end of LDs indicates that AFR1/AFR2-HDAC may accumulate at dusk, which may enable or enhance their recruitment by AGL18 to the *FT* proximal promoter. CO activity not only enables AGL18-AFR-HDAC binding to *FT* chromatin to dampen *FT* expression, but may also facilitate dynamic recruitment of a HAT to the *FT* locus to promote its expression given the dynamic nature of histone acetylation and deacetylation on *FT* chromatin at dusk (Figure 8). The opposing activities of HAT and AFR-HDAC at the end of LDs conceivably modulate the acetylation dynamics of *FT* chromatin and set *FT* expression at an adequate level at the right time.

### Role of AFR-HDAC for Photoperiodic Flowering-Time Regulation

We show here that *AFR*s act in the photoperiod pathway to dampen *FT* expression in response to inductive long days and so prevent precocious flowering in *Arabidopsis*. Interestingly, only a moderate early-flowering phenotype has been observed in the *afr1 afr2* mutant in which *AFR1* is still expressed at a low level (Figure S3A). It is likely that a complete loss of function of *AFR1* and *AFR2* may cause a strong early-flowering phenotype. In short days, the CO protein does not accumulate and thus *FT* is expressed at a very low level [2]. We have observed that in short days *FT* expression is relatively high at ZT4 and ZT20-ZT24 in WT, and that loss of *AFR1* and *AFR2* function leads to a moderate *FT* upregulation specifically at these time points (Figure S7B). In addition, it has been shown that *AGL18* also moderately represses *FT* expression in short days [35]. Taken together, these findings suggest that AGL18-AFR-HDAC acts to downregulate *FT* expression to prevent early flowering in non-inductive short days as well, conferring an accurate photoperiodic response.

*CO* and *FT* are widely conserved among angiosperms from the monocotyledonous rice grass to the dicotyledonous poplar tree [1],[2]. In the plants so far examined, the function of *FT* or *FT* homologs as a major inducer of flowering (florigen) is largely conserved, and the *CO-FT* regulatory module underlying the photoperiodic flowering-time control in the dicotyledonous LD plant *Arabidopsis*, also plays a central role for flowering-time control in the monocotyledonous short-day plant rice [2]. Homologs of the core components of AFR-HDAC including AFR1/AFR2, AtSAP18, RPD3-type HDACs, and SNLs are highly conserved in angiosperms (Figure S1B) [22],[23],[25]. These findings collectively indicate that the temporal chromatin mechanism of periodic histone deacetylation underlying *FT* expression control in *Arabidopsis* may modulate the expression of *FT* homologs in other plant species and so confer an optimal flowering time in response to photoperiodic changes.

## Materials and Methods

### Plant Materials and Growth Conditions

The *co*, *ft*, *fve*, *agl15-3*, and *agl18-1* were described previously [6],[27],[35],[41]. *afr1-1* (Salk_110828), *afr1-2* (Salk_059944), *afr2-1* (Salk_032653), and *afr2-2* (CS842844) were obtained from the *Arabidopsis* Biological Resource Center and directly used in this study. Plants were grown in long days (16-h light/8-h dark) or short days (8-h light/16-h dark) under cool white fluorescent light at 22°C [41].

### Yeast Two-Hybrid Assay

The Matchmaker GAL4 Two-Hybrid System 3 (Clontech) was adapted for this assay. Full-length coding sequences for AFR1, AFR2, AtSAP18, HDA9, HDA19, and SNL2 were cloned into the *pGADT7* and/or *pGBKT7* vectors, which were subsequently introduced into the yeast strain *AH109* according to the manufacturer's instructions (Clontech). Yeast cells were spotted on selective media lacking of leucine, tryptophan, and histidine.

### BiFC Assay

The full-length coding sequences for AFR1, AFR2, AtSAP18, HDA19, AGL15, and AGL18 were fused in frame with either the coding sequence for an N-terminal EYFP fragment in the *pSAT1A-nEYFP-N1/pSAT1-nEYFPC1* vectors and/or for a C-terminal EYFP fragment in the *pSAT1AcEYFP-N1/pSAT1-cEYFP-C1-B* vectors [42]. Plasmid pairs were used to transiently transform onion epidermal cells via bombardment by the Helium Biolistic Gene Transformation System (Bio-Rad). Within 24 h following the bombardment, the EYFP fluorescence emitted from the onion cells was imaged with a Zeiss LSM 5 EXCITER upright laser scanning confocal microscopy (Zeiss) [43].

### RNA Analysis by Real-Time Quantitative PCR

Total RNAs were extracted from aerial parts of 10- to 11-d-old seedlings grown in long days (at various time points) or 21-d-old seedling grown in short day using the RNeasy Plus Mini Kit (Qiagen) according to the manufacturer's instructions. cDNAs were reverse transcribed from the total RNAs. Real-time quantitative PCR (qRT-PCR) was performed on an ABI Prism 7900HT sequence detection system using a SYBR green PCR master mix; PCR was conducted as follows: 50°C (2 min), 95°C (10 min), and 40 cycles of 95°C (15 s) and 60°C (60 s). Each sample was quantified at least in triplicate, and the constitutively expressed *UBQ10* (its expression is not influenced by day-night cycles) [44], was used for normalization. Ratio of the transcript level of a gene of interest to that of *UBQ10* is calculated as 2^−ΔCt^ [ΔC_T_ = C_T_ (gene of interest) − C_T_ (*UBQ10*)]. The primer pair for *CO* amplification has been described previously [44], and primer pairs for *AFR1*, *AFR2*, *FT*, and *UBQ10* amplifications are specified in Table S1.

### Plasmid Construction

To construct *AFR1-GUS* and *AFR2-GUS*, genomic fragments of 3.0-kb *AFR1* (1.0-kb 5′ promoter plus 2.0-kb genomic coding sequence) and 3.9-kb *AFR2* (1.2-kb 5′ promoter plus 2.7-kb genomic coding sequence) were inserted upstream of *GUS* in the *pMDC162* vector [45] via gateway technology (Invitrogen). For AFR1 and AFR2 subcellular localization, the full-length *AFR1* and *AFR2* coding sequences (except the stop codons) were inserted between the *35S* promoter and *GFP* in the *pMDC85-GFP* vector [45] via gateway technology (*AFR1* and *AFR2* are in frame with *GFP*).

To generate the *pAFR1-AFR1:HA* plasmid, a 3.0-kb *AFR1* genomic fragment (1.0-kb 5′ promoter plus the 2.0-kb full-length genomic coding sequence except the stop codon) was first fused with a *3xHA* tag, and cloned into the *pHGW* vector [46] via gateway technology. For *pAFR2-AFR2:FLAG* construction, a 3.9-kb *AFR2* genomic fragment (1.2-kb 5′ promoter plus the 2.7-kb full-length genomic coding sequence except the stop codon) was first fused with a *3xFLAG* tag, and cloned into *pHGW*. To construct *p35S-AGL18:FLAG*, the full-length *AGL18* coding sequence without the stop codon (772 bp) was first fused with a *3xFLAG* tag, and the *AGL18:FLAG* fusion was subsequently placed downstream of the *35S* promoter in the *pB2GW7* vector [46] via that gateway technology. The sequences of primers used for plasmid construction are specified in Table S1.

### Co-immunoprecipitation

Co-immunoprecipitation experiments were carried out as previously described [43]. Briefly, total proteins were extracted from 10-d-old seedlings and immunoprecipitated with anti-HA M2 affinity gel (Sigma, catalog number A2220). AGL18:FLAG in the immunoprecipitates was detected by western blotting with anti-FLAG (Sigma, catalog number A8592).

### Chromatin Immunoprecipitation

ChIP experiments were carried out as described previously with minor modifications [47]. Briefly, total chromatin was extracted from 10-d-old seedlings or the first pair of rosette leaves from 13-d-old seedlings grown in LDs, and immunoprecipitated with Rabbit polyclonal anti- acetylated histone H3 (with acetyl K9+K14+K18+K23+K27; abcam, catalog number ab47915), anti-HA (Sigma, catalog number H6908), or anti-FLAG (Sigma, catalog number F7425). Quantitative PCR was conducted to measure the amounts of *FT* and the constitutively expressed *TUB2* and *TUB8* fragments on an ABI Prism 7900HT sequence detection system using a SYBR Green PCR master mix. The primers used are specified in Table S1.

## Supporting Information

Figure S1 **Alignments of AFR1 and AFR2 relatives.** Numbers refer to amino acid residues. Identical residues among these proteins are shaded black, whereas similar residues are shaded gray. (A) Alignment of the *Arabidopsis* AFR1 (AtAFR1) and AFR2 (AtAFR2) with the yeast Sap30 (ScSap30). The conserved residues in the putative SBRs are indicated by asterisks (*). (B) Alignment of AtAFR1 with its homologs from other plants including *Populus trichocarpa* (PtSFR), *Picea sitchensis* (PsSFR), and *Physcomitrella patens* (PpSFR). The GenBank accession numbers for PtSFR, PsSFR, and PpSFR are XP_002302473.1, ADE76839.1, and XP_001766227.1, respectively.
(EPS)

Figure S2 **Direct interactions of AT1G75060 (AFR1) and AT1G19330 (AFR2) with SNL2 and HDA9 proteins in yeast.** The indicated full-length proteins were fused with the GAL4-BD or AD domains. Yeast cells harboring the fusion proteins, BD and/or AD (as indicated), were grown on the selective synthetic defined media lacking of Trp (W), Leu (L), and His (H). (A,B) Interactions of SNL2 with AFR1 and AFR2 in yeast. (C,D) Interactions of HDA9 with AFR1 and AFR2 in yeast.
(TIF)

Figure S3 **Analysis of** ***AFR1*** **and** ***AFR2*** **expression in** ***afr1*** **and** ***afr2*** **seedlings by semiquantitative RT-PCR.** The constitutively expressed *TUB2* served as a control. (A) Analysis of the full-length *AFR1* and *AFR2* transcripts. (B) Analysis of 5′- or 3′-truncated *AFR2* transcripts in *afr2* mutants.
(EPS)

Figure S4 **Rescue of** ***afr1*** **and** ***afr2*** **mutants by** ***pAFR1-AFR1:HA*** **and** ***pAFR2-AFR2:FLAG*** **, respectively.** Plants were grown in short days, and total leaf number for each line (9–11 plants per line) was scored. Bars indicate SD. (A) Flowering times of *afr1-1* and a transgenic line of *pAFR1-AFR1:HA* in *afr1-1* (native *AFR1* fused with 3x *HA*; single-locus T_3_ homozygotes). (B) Flowering times of *afr2-1* and a transgenic line of *pAFR2-AFR2:FLAG* in *afr2-1* (native *AFR2* fused with 3x *FLAG*; single-locus T_3_ homozygotes).
(EPS)

Figure S5 **Analysis of flowering time and leaf initiation rate of** ***afr1 afr2*** **in LDs.** 12 plants were scored for each line; bars for SD. (A) Days to flowering of Col and *afr1 afr2*. (B) Total leaf numbers at flowering of Col and *afr1 afr2*. (C) Leaf initiation rates of Col and *afr1 afr2* plants.
(EPS)

Figure S6 **Relative** ***AFR1*** **and** ***AFR2*** **transcript levels in Col and** ***co*** **seedlings at the end of LDs, quantified by qRT-PCR.** The transcript levels were normalized first to the endogenous control *UBQ10*, and relative fold changes to Col are presented. Bars indicate SD of triplicate measurements.
(EPS)

Figure S7 ***AFR1*** **and** ***AFR2*** **moderately repress** ***FT*** **expression in short days.** (A) Flowering times of the indicated lines grown in short days. Total leaf number for each line (8–14 plants per line) was scored. Error bars indicate SD. (B) *FT* mRNA levels in Col and *afr1 afr2* seedlings over a 24-h short-day cycle. Total RNAs were extracted from 21-d old seedlings every 4 h and quantified by qRT-PCR. Bars indicate SD of three measurements.
(EPS)

Figure S8 **Biological repeats of** ***FT*** **,** ***AFR1*** **, and** ***AFR2*** **expression analyses.** (A) A biological repeat of the *FT* expression analysis presented in Figure 3E. (B) A biological repeat of the *AFR1* and *AFR2* expression analysis presented in Figure 4C.
(EPS)

Figure S9 **Relative levels of AFR1:HA and AFR2:FLAG proteins over a 24-h LD cycle.** The proteins in respective 10- to 11-d-old seedlings were analyzed by western blotting. The intensities of protein bands were quantified by the ImageJ 1.44j program, and relative levels to ZT0 are presented. Note that the time of the day has a significant effect on the levels of both AFR1 and AFR2 proteins in the seedlings, as revealed by single-factor ANOVA tests (for AFR1, *p*<0.01; for AFR2, *p*<0.05). (A) Relative AFR1:HA levels over a 24-h LD cycle. Average values of three biological repeats are presented; bars for SD. (B) Relative AFR2:FLAG levels over a 24-h LD cycle. Average values of four biological repeats are presented; bars for SD.
(EPS)

Figure S10 **Biological repeats of the ChIP assays on AFR1 and AFR2 binding to** ***FT*** **chromatin.** (A) A biological repeat of the ChIP analysis presented in Figure 4E. (B) A biological repeat of the ChIP analysis presented in Figure 4F. (C) A biological repeat of the ChIP analysis presented in Figure 7.
(EPS)

Figure S11 **ChIP analysis of the histone H3 acetylation state of** ***FT*** **chromatin in Col and** ***afr1 afr2*** **seedlings grown in LDs.** Amounts of the immunoprecipitated genomic DNA was quantified and normalized first to the constitutively expressed *TUB2*. Subsequently, the fold enrichments were calculated by dividing the levels of acetylated H3 in *afr1 afr2* (at ZT8 or ZT16) or the WT at ZT16, by the level in the WT at ZT8. Shown are the means and SD of two ChIP experiments.
(EPS)

Figure S12 **Direct interactions of AGL18 with AFR2 and AGL15 with AFR1.** (A) BiFC analysis of the interaction of AGL18 with AFR2 in onion epidermal cells. Yellowish-green signals indicate the binding of AGL18 with AFR2 in the nuclei (indicated by the blue fluorescence from DAPI). Bar = 20 µm. (B) AFR1 interacts with AGL15, but not FLC, in yeast cells. The full-length AFR1, and full-length FLC and the 208-aa AGL15 (without MADS domain) were fused with the GAL4-AD and BD domains, respectively. Yeast cells were grown on the selective synthetic defined media lacking of W, L, and H or lacking of W and L. Of note, FLC directly interacts with SVP and binds to *FT* chromatin to repress *FT* expression [48], and serves as a negative control in this experiment. (C) BiFC analysis of the interaction of AGL15 with AFR1 in onion epidermal cells. The full-length AGL15 and AFR1 were fused with nEYFP and cEYFP fragments, respectively. Yellowish-green signals indicate the binding of AGL15 with AFR1 in the nuclei. Bar = 20 µm.
(TIF)

Figure S13 **Characterization of the** ***p35S-AGL18:FLAG*** **line and a biological repeat of ChIP analysis of AGL18 binding to** ***FT*** **chromatin.** (A) Flowering times of the *p35S-AGL18:FLAG* line (T_3_; in the Col background) grown in LDs. Overexpression of *AGL18* causes moderate late flowering [35]. Double asterisk indicates a statistically significant difference in the means between Col and the transgenic line, as revealed by two-tailed Student's *t* test (**, *p*<0.01). Bars indicate SD. (B) A biological repeat of ChIP analysis of AGL18 binding to *FT* chromatin presented in Figure 6E.
(EPS)

Figure S14 **Analysis of** ***AGL15*** **and** ***AGL18*** **expression in Col and** ***agl15 agl18*** **seedlings by RT-PCR.** The constitutively expressed *TUB2* served as a control.
(EPS)

Figure S15 **Examination of CO interaction with AFR1, AFR2, or AGL18 by the yeast-two-hybrid assay.** The full-length CO was fused with GAL4-AD, whereas the full-length AFR1, AFR2, and AGL18 were fused with GAL4-BD. Yeast cells were grown on the selective synthetic defined media lacking of W, L, and H or lacking of W and L.
(TIF)

Table S1 **List of primers used in this study.**
(DOCX)

## Abbreviations

AFR1: SAP30 FUNCTION-RELATED 1
AFR2: SAP30 FUNCTION-RELATED 2
AGL18: AGAMOUS LIKE 18
BiFC: bimolecular fluorescence complementation
ChIP: chromatin immunoprecipitation
CO: CONSTANS
EYFP: enhanced yellow fluorescent protein
FT: FLOWERING LOCUS T
GUS: β-GLUCURONIDASE
HAT: histone acetyltransferase
HDAC: histone deacetylase
LD: long day
qRT-PCR: real-time quantitative PCR
RPD3: Reduced Potassium Dependency-3
SAM: shoot apical meristem
SAP30: Sin3-Associated Polypeptide 30
SBR: Sin3-binding region
SD: standard deviation
,: Sin3-Associated Polypeptide 18 (SAP18)
ZT: Zeitgeber time
