# Supplementary figures and images for: Photoperiodic Regulation of Flowering Time through Periodic Histone Deacetylation of the Florigen Gene FT
Source: PLoS Biol. 2013 Sep 3;11(9):e1001649. doi: 10.1371/journal.pbio.1001649 (PMC3760768; doi:10.1371/journal.pbio.1001649)

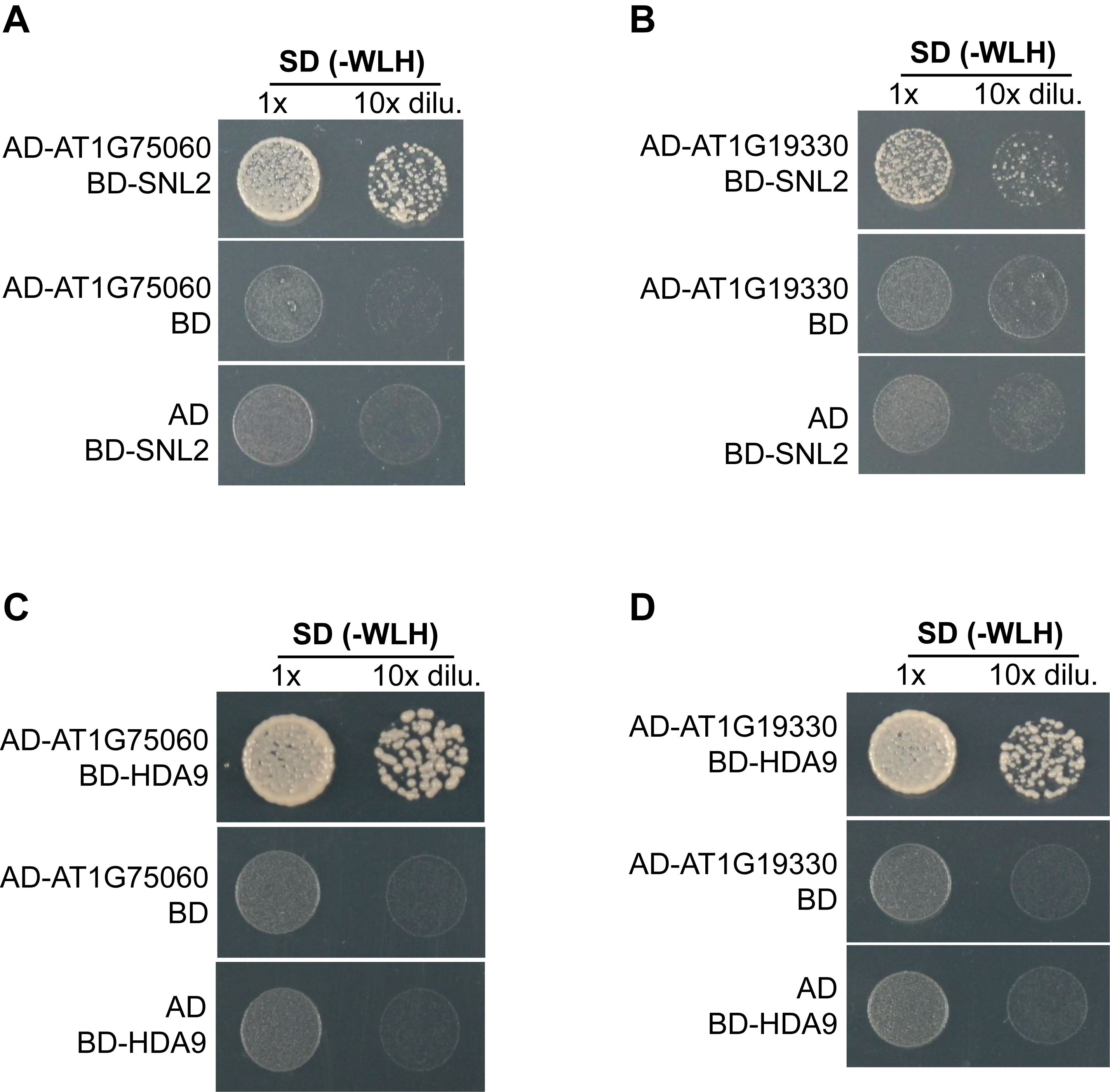

Supplement: Figure S2 — Direct interactions of AT1G75060 (AFR1) and AT1G19330 (AFR2) with SNL2 and HDA9 proteins in yeast. The indicated full-length proteins were fused with the GAL4-BD or AD domains. Yeast cells harboring the fusion proteins, BD and/or AD (as indicated), were grown on the selective synthetic defined media lacking of Trp (W), Leu (L), and His (H). (A,B) Interactions of SNL2 with AFR1 and AFR2 in yeast. (C,D) Interactions of HDA9 with AFR1 and AFR2 in yeast. (TIF) [file pbio.1001649.s002.tif]

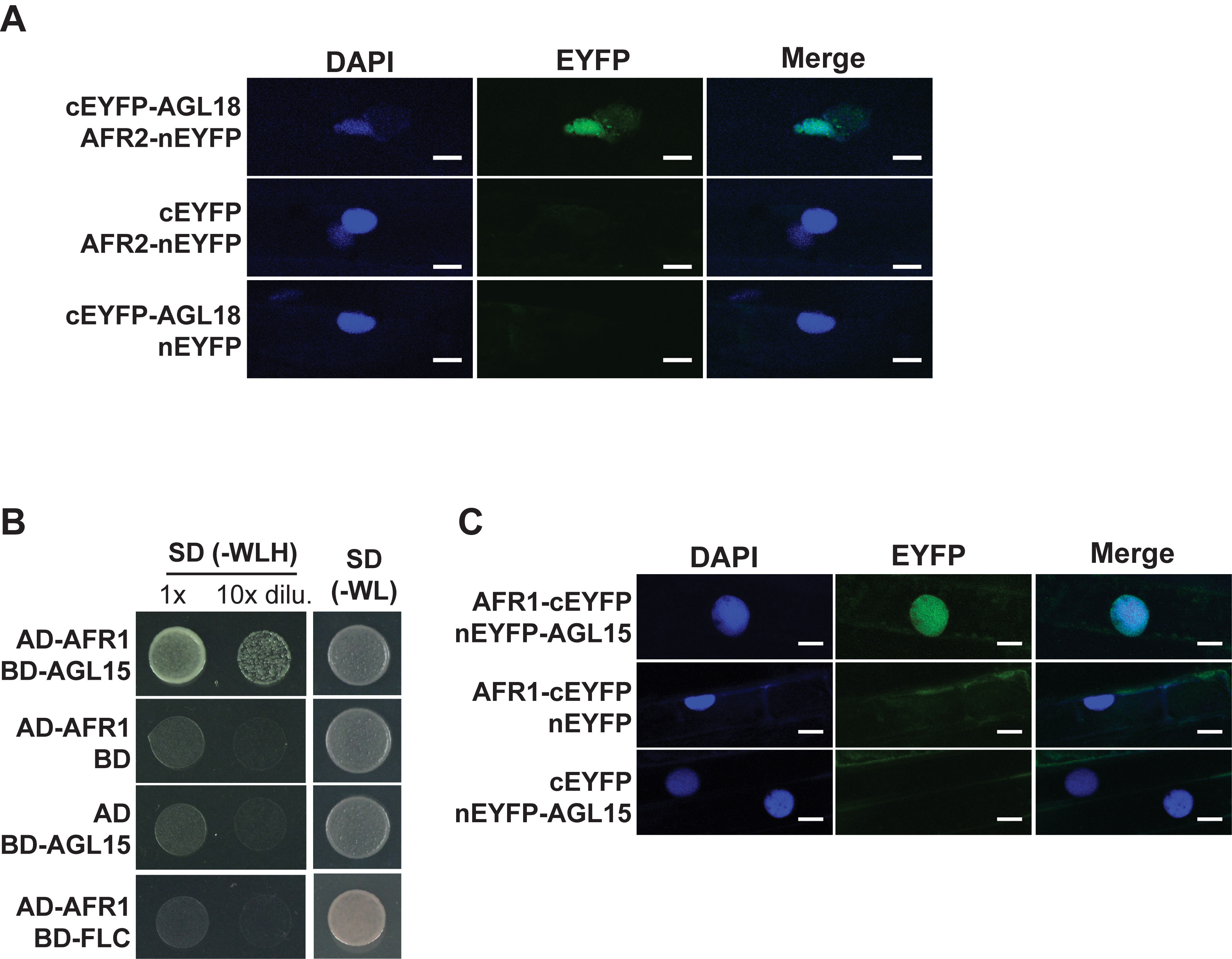

Supplement: Figure S12 — Direct interactions of AGL18 with AFR2 and AGL15 with AFR1. (A) BiFC analysis of the interaction of AGL18 with AFR2 in onion epidermal cells. Yellowish-green signals indicate the binding of AGL18 with AFR2 in the nuclei (indicated by the blue fluorescence from DAPI). Bar = 20 µm. (B) AFR1 interacts with AGL15, but not FLC, in yeast cells. The full-length AFR1, and full-length FLC and the 208-aa AGL15 (without MADS domain) were fused with the GAL4-AD and BD domains, respectively. Yeast cells were grown on the selective synthetic defined media lacking of W, L, and H or lacking of W and L. Of note, FLC directly interacts with SVP and binds to FT chromatin to repress FT expression [48], and serves as a negative control in this experiment. (C) BiFC analysis of the interaction of AGL15 with AFR1 in onion epidermal cells. The full-length AGL15 and AFR1 were fused with nEYFP and cEYFP fragments, respectively. Yellowish-green signals indicate the binding of AGL15 with AFR1 in the nuclei. Bar = 20 µm. (TIF) [file pbio.1001649.s012.tif]

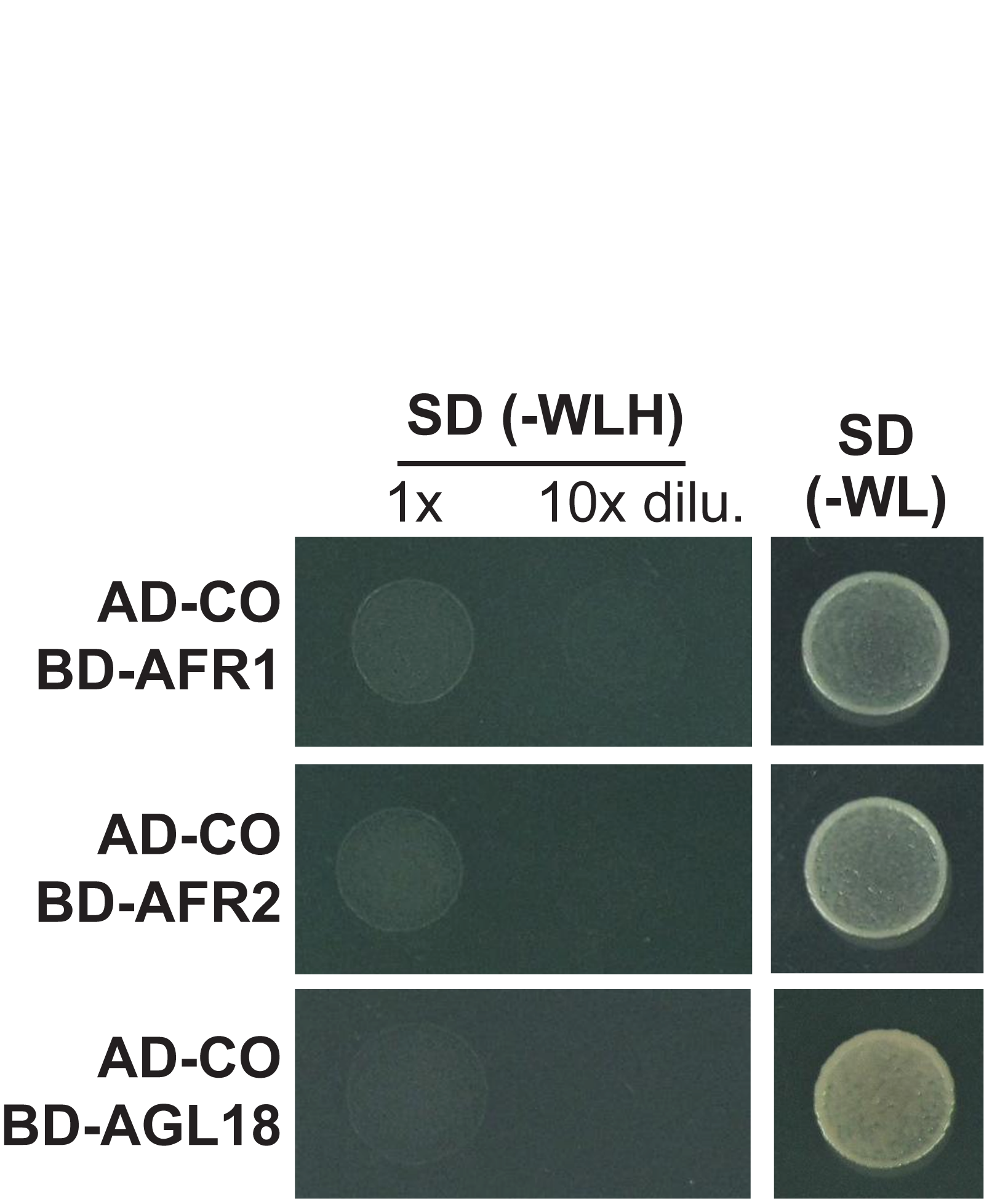

Supplement: Figure S15 — Examination of CO interaction with AFR1, AFR2, or AGL18 by the yeast-two-hybrid assay. The full-length CO was fused with GAL4-AD, whereas the full-length AFR1, AFR2, and AGL18 were fused with GAL4-BD. Yeast cells were grown on the selective synthetic defined media lacking of W, L, and H or lacking of W and L. (TIF) [file pbio.1001649.s015.tif]
