# Supplementary material for: Photoperiodic Regulation of Flowering Time through Periodic Histone Deacetylation of the Florigen Gene FT
Source: PLoS Biol. 2013 Sep 3;11(9):e1001649. doi: 10.1371/journal.pbio.1001649 (PMC3760768; doi:10.1371/journal.pbio.1001649)
Supplement: Table S1 — List of primers used in this study. (DOCX) [file pbio.1001649.s016.docx]

**Table S1.** **List of primers used in this study**

Experiments Amplified regions Forward (F) and reverse (R) primers

**RT-qPCR** *AFR1* F: GGAAGTTTCTTGTGACTCACACTCAA

R: CCTGAACGAATCCCACAATAACC

*AFR2* F: ATTGTTTCTTTTCCAGCATCTGAGG

R: CTTTGATGGATTTGGAATTGCATC

*FT* F: GACCTCAGGAACTTCTATACTTTGGTTATG

R: CTGTTTGCCTGCCAAGCTG

*UBQ10* F: AAATCTCGTCTCTGTTATGCTTAAGAAG

R: TTTTACATGAAACGAAACATTGAACTT

**ChIP-qPCR** *FT-P* F: CCAGTGTATTAGTGTGGTGGGTTTG

R: GCATTAACTCGGGTCGGTGAAATC

*FT-E1* F: GAGACCCTCTTATAGTAAGCAGAGTTG

R: GGGAGTTCAAGTGAAAGAACCAAAGT

*FT-I1* F: CTTGGGATTTTTCTTTGTTCCTCCTACC

R: CAGTTTTCAAATATTCCACAACAGAGATTCATC

*TUB2* F: ATCCGTGAAGAGTACCCAGAT

R: AAGAACCATGCACTCATCAGC

*TUB8* F: GTCATAACCGTTTCAAATTCTCTCTCTCT

R: TGCAAATCGTTCTCTCCTTGGTATC

**Plasmid** *AFR1*  F: GCGGGATCCAATTATTGGTGATACGTTTTAAATCATCAGTT

**construction** R: GGGGCGGCCGCGTGTTTCTGGCTTCTTTGGTCTTCC

(For *pAFR1-AFR1:GUS* and *pAFR1-AFR1:HA*)

*AFR2* F: GCGGTCGACTAGAGACAGACGTGTAGGATTATTCTCATTAACC

R: GGGGCGGCCGCGCTGATGCAGTTAAGATCAGTGTTTCTGG

(For *pAFR2-AFR2:GUS* and *pAFR2-AFR2:FLAG*)

*AFR1* F: GCGGGATCCAGAGAGAGAGAAAACACAAAATGCTGG

(For *AFR1:GFP*) R: Same as the reverse primer for *pAFR1-AFR1:GUS*

*AFR2* F: GCGGTCGACAAGAAGAAGAAGAATGCTGGAAGCTGTAG

(For *AFR2:GFP*) R: Same as the reverse primer for *pAFR2-AFR2:GUS*

*AGL18* F: CGGCCATGGAGAGATATGGGGAGAGGAAGGATTGAG

(*p35S-AGL18:FLAG*) R: CGGCTCGAGATCAGAAGCCACTTGACTCCCAG
